# Generation of murine macrophage-derived cell lines expressing porcine CD163 that support porcine reproductive and respiratory syndrome virus infection

**DOI:** 10.1186/s12896-017-0399-5

**Published:** 2017-11-09

**Authors:** Liangliang Li, Chunyan Wu, Gaopeng Hou, Biyun Xue, Sha Xie, Qin Zhao, Yuchen Nan, Gaiping Zhang, En-Min Zhou

**Affiliations:** 1 Department of Preventive Veterinary Medicine, College of Veterinary Medicine, Northwest A&F University, Yangling, Shaanxi China; 2 Scientific Observing and Experimental Station of Veterinary Pharmacology and Diagnostic Technology, Ministry of Agriculture, Yangling, Shaanxi China; 3 College of Animal Science and Veterinary Medicine, Henan Agricultural University, Zhengzhou, Henan China

**Keywords:** Murine macrophage-derived cells, Porcine CD163, PRRSV

## Abstract

**Background:**

Porcine reproductive and respiratory syndrome virus (PRRSV) exhibits a highly restricted tropism for cells of the monocyte-macrophage lineage, utilizing porcine CD163 (pCD163) as an indispensable cellular receptor for infection. Transfection the gene of pCD163 into several non-permissive cell lines followed by protein expression confers susceptibility to PRRSV. A lack of specialized porcine antibody tools for use with existing porcine-derived primary cells and cell lines has hampered studies of both PRRSV pathogenesis and virus triggering of immune response cascades. Therefore, we constructed PRRSV-susceptible murine alveolar macrophage-derived MH-S and peritoneal macrophage-like RAW264.7 cell lines by achieving pCD163 cell surface expression in these cells. We then evaluated PRRSV susceptibility and cytokine expression patterns induced upon PRRSV infection of these pCD163-expressing cell lines.

**Results:**

Growth of MH-S^CD163^ and RAW264.7^CD163^ cells was indistinguishable from growth of un-transfected parental cell lines. Meanwhile, various stages of the PRRSV replication cycle, including viral particle attachment, internalization, disassembly and infection were confirmed in both pCD163-transfected cell lines. Analysis of PRRSV replication using immunofluorescence staining of virus and viral titration of cell lysates demonstrated that both MH-S^CD163^ and RAW264.7^CD163^ cells supported replication of various genotype 2 PRRSV isolates. Moreover, PRRSV replication in MH-S^CD163^ cells was similar to that observed in porcine alveolar macrophages (PAMs) and was more efficient than in RAW264.7^CD163^ cells. However, peak virus titers in MH-S^CD163^ cells were attained at 60 h post-infection (pi) versus 48 hpi in PAMs. Analysis of cytokine expression showed that post-PRRSV infection, mRNA expression patterns of anti-inflammatory cytokines (IL-4 and IL-10) and pro-inflammatory cytokines (TNF-α and IFN-γ) in MH-S^CD163^ cells were more similar to those observed in PAMs versus levels in RAW264.7^CD163^ cells.

**Conclusions:**

MH-S and RAW264.7 cells were not susceptible to PRRSV infection until transfection and subsequent expression of pCD163 were achieved in these cell lines. The PRRSV-susceptible MH-S^CD163^ cell line efficiently supported viral replication of various genotype 2 PRRSV isolates and exhibited similar cytokine expression patterns as observed in PAMs. In conclusion, this work describes the development of new tools to further understand PRRSV pathogenesis and immune response mechanisms to PRRSV infection.

**Electronic supplementary material:**

The online version of this article (10.1186/s12896-017-0399-5) contains supplementary material, which is available to authorized users.

## Background

Porcine reproductive and respiratory syndrome is a major swine infectious disease that causes severe economic losses for the global swine industry [1]. Its etiologic agent, porcine reproductive and respiratory syndrome virus (PRRSV), is an enveloped virus member of the family *Arteriviridae*, order *Nidovirales*, with a positive-stranded RNA genome [2, 3]. There are two PRRSV genotypes; PRRSV-1 (genotype first isolated in Europe) and PRRSV-2 (genotype first isolated in North America) [4, 5]. These two genotypes share approximately 60% sequence identity but differ significantly serotype and virulence [6]. The PRRSV genome contains nine open reading frames (ORFs). ORFs 1a and 1b comprise 80% of the viral genome and are predicted to encode the necessary enzymes for viral RNA replication. PRRSV ORFs 2, 2a, 3-7 and 5a encode eight known structural proteins, which are minor membrane-associated proteins GP2, E, GP3 and GP4, a major envelope glycoprotein (GP5), a membrane protein (M), a nucleocapsid protein (N) and a novel ORF5a-encoded protein [7–9].

Unlike other arteriviruses, which have a relatively broad cell tropisms [10], PRRSV has a highly restricted tropism for cells of the monocyte-macrophage lineage, specifically porcine alveolar macrophages (PAMs), during acute infection of pigs [11, 12]. Currently, PRRSV can only be propagated *in vitro* in epithelial-derived MARC-145 cells, a subclone of the African green monkey kidney cell line MA104 [13]. Other cell lines, such as porcine kidney (PK-15), baby hamster kidney cells (BHK-21) and a PAM-derived cell line (CRL-2843) expressing exogenous porcine CD163 (pCD163) are capable of PRRSV infection [14–16]. However, the lack of specialized antibodies recognizing immunologic proteins of porcine origin (e.g., swine cluster of differentiation (CD) antigens and swine leukocyte antigens), has significantly hampered further research on PRRSV pathogenesis mechanisms and virus-triggered immune response cascades in porcine-derived primary cells or cell lines.

To date, host factors involved in the PRRSV cellular tropism are still not fully understood. Numerous *in vitro* studies have demonstrated that PRRSV infection is determined by various cellular receptors or factors [17] that include heparin sulfate (HS) [18], vimentin [19], CD151 [20], pCD163 [21], sialoadhesin (CD169) [22], DC-SIGN (CD209) [23] and MYH9 [24]. With the development of *in vivo* genetic engineering technology, recent studies with the gene knocked-out pigs demonstrate that pCD163 [25] but not CD169[26] is indispensable for successful infection with PRRSV.

In this study we introduced pCD163 into a Balb/c J mouse bronchoalveolar macrophage-derived MH-S cell line which undergoes immortalization via introduction of SV40-LT antigen [27], and a mouse macrophage-like RAW264.7 cell line was derived from a murine leukemia virus (MuLV)-transformed tumor and is free of replication-competent MuLV [28, 29], both of which have been widely used to evaluate macrophage-specific immune responses *in vitro* [30, 31]. Our results demonstrated that MH-S and RAW264.7 cell lines stably expressed pCD163 (designated MH-S^CD163^ and RAW264.7^CD163^, respectively) and supported infection and replication of various genotype 2 PRRSV isolates. Virus titers in MH-S^CD163^ cells were similar to that observed in primary PAMs and were even higher than in RAW264.7^CD163^ cells. Moreover, PRRSV-induced cytokine expression patterns in MH-S^CD163^ cells more closely mirrored patterns observed in PAMs than that observed in RAW264.7^CD163^ cells. Taken together, our findings provide new tools for further research to elucidate PRRSV pathogenesis and cellular immune response mechanisms to PRRSV infection.

## Methods

### Cells and viruses

A mouse alveolar macrophage-derived cell line MH-S, a peritoneal macrophage-like cell line RAW264.7 and MARC-145 cells were purchased from the China Center for Type Culture Collection (CCTCC, Wuhan, China). Primary PAMs were prepared from bronchoalveolar lavage of 4 to 6-week-old PRRSV-negative piglets. Culture and preparation of PAMs were conducted as previously described [32, 33]. PAMs and the MH-S cell line were maintained in RPMI 1640 (Gibco, Carlsbad, CA, USA) supplemented with 10% FBS (v/v; BI, Israel). RAW264.7 and MARC-145 cell lines were cultured in Dulbecco’s Modified Eagle Medium (DMEM) (Gibco) containing 10% fetal bovine serum (FBS) (BI).

Various genotype 2 PRRSV isolates including highly pathogenic PRRSV strains (listed with Genbank accession numbers in parentheses), JXA1 (GenBank: EF112445.1), SD16 (GenBank: JX087437.1), GD-HD (GenBank: KP793736.1) and classical strain VR-2332 (GenBank: AY150564 ) were used to infect the various cell lines at 0.1 to 10 multiplicity of infection (MOI). Viral titers were determined in MARC-145 cells by calculating the median tissue culture infective dose (TCID_50_) as previously described [34].

### Transfection vector construction, lentiviral particle preparation and cell transduction

The cDNA fragment encoding pCD163 (GenBank: JX292263) was amplified from total RNA isolated from PAMs using pCD163-F/pCD163-R primers (Table 1) and ligated into the pTRIP-CMV-Puro lentiviral vector [35] to generate the pTRIP-CMV-Puro-pCD163 construct. Successful insertion of targeting cDNA was verified using DNA sequencing (Sangon Biotech Inc., Shanghai, China).

**Table 1:** Primer list for full-length of genome amplification or cytokines expression

Genes	Forward primer	Reverse primer	References
CD163	GCTCTAGAATGGTGCTACTTGAAG	CGGGATCCTCATTGTACTTCAGAGTGG	Xiangpeng wang et al. 2013
ORF7	ATGCCAAATAACAACGGCAAGCAGC	TCATGCTGAGGGTGATGCTGTG	Xiangpeng wang et al. 2013
GAPDH	CCTTCCGTGTCCCTACTGCCAAC	GACGCCTGCTTCACCACCTTCT	Xin-xin Chen et al. 2014
TNFα	^a^ AACCTCAGATAAGCCCGTCG ^b^ GGCAGGTCTACTTTGGAGTCAT	ACCACCAGCTGGTTGTCTTT CAGAGTAAAGGGGTCAGAGTGG	Gudmundsdottir and Risatti 2009 Tingyu Wang et al. 2014
IL4	^a^ GCCGGGCCTCGACTGT ^b^ CATCGGCATTTTGAACGAG	TCCGCTCAGGAGGCTCTTC TGGAAGCCCTACAGACAAGC	Dawson et al. 2005
IL10	^a^ CGGCGCTGTCATCAATTTCTG ^b^ GGACAACATACTGCTAACCGACT	CCCCTCTCTTGGAGCTTGCTA TGGGGCATCACTTCTACCA	Duvigneau et al. 2005
IFNγ	^a^AATGGTAGCTCTGGGAAACTG ^b^ TGC TGA TGG GAG GAG ATGTCT	ACTTCTCTTCCGCTTTCTTAGG TGC TGT CTG GCC TGC TGT TA	Yoo Jin Lee et al. 2012; Yinhang Yu et al.2016
TGFβ	^a^ CGCCTGCTGAGGCAAAGT ^b^ TGACGTCACTGGAGTTGTACGG	GAGGTAGCGCCAGGAATCATT GGTTCATGTCATGGATGGTGC	Min Song et al. 2011

*IL* interleukin, *TNF* tumor necrosis factor, *TGF* transforming growth factor; ^a^primers of swine-origin; ^b^primers of mouse-origin

Recombinant lentiviral particles carrying pCD163 were obtained by co-transfection of three kinds of vector (pTRIP-CMV-Puro-CD163, pMD2.G and psPAX2) into HEK293T cells using the X-tremeGENE^TM^ HP DNA Transfection Reagent (Roche, Switzerland) according to the manufacturer’s instructions; packed empty lentiviral particles served as the control. Cell culture supernatants containing lentiviral particles were harvested 48 h post-transfection.

The pCD163-expressing cell lines, MH-S^CD163^ and RAW264.7^CD163^, were established using transduction of recombinant lentiviruses followed by puromycin selection (30 μg/ml, Merck, USA). Subcloning of surviving cells was done using limiting dilution in 96-well plates. The MH-S and RAW264.7 cells transduced with empty vector were designated MH-S^vector^ and RAW264.7^vector^ and served as control cell lines.

### Cell proliferation assay

Cell proliferation of the various cell lines was assessed as previously described [36, 37] with the following modifications. Briefly, MH-S and RAW264.7 cells transduced with lentiviral particles carrying pCD163 or empty vector, as well as their parental un-transfected cells, were seeded in 24-well plates at a density of 1×10^4^ cells/well and were trypsinized daily for a total of eight consecutive days whereby each day half of the cells from each well were removed and counted to determine total cell numbers for evaluation of proliferation rates.

### Analysis of PRRSV attachment, internalization, disassembly and infection

PRRSV attachment, internalization, disassembly and infection of the various cell lines were assayed as previously described [14] with the following modifications. Briefly, MH-S^CD163^ and RAW264.7^CD163^ cell lines and PAMs were incubated with PRRSV strains JXA1 or VR-2332 at a MOI of 10. Each cell type was analyzed for each of four stages of virus infection using four replicate cell cultures. For visualization of virus attachment, one replicate cell culture was fixed with 4% paraformaldehyde after 1 h of incubation with virus at 4°C. To observe virus internalization, a second cell replicate culture was incubated at 37°C with virus for 1 h then the cells were fixed and permeabilized for virus visualization. A third cell replicate culture for each treatment was incubated as for the second replicate, but was incubated at 37°C for an additional 4 h and then fixed to measure disassembled viral particles. The fourth replicate cell culture was analyzed by fixation of cells after incubation with virus for 24 h at 37°C. For visualization of virus infection at each stage, virus was detected using a mouse monoclonal antibody against PRRSV N protein (Clone No. 6D10, in house) as previously described [35] and labeled secondary antibody described below.

### Western blot

Sodium dodecyl sulfate-polyacrylamide gel electrophoresis (SDS-PAGE) and western blot were conducted as previously described [38, 39] with the following modifications. Briefly, after cells were lysed using NP40 cell lysis buffer (Beyotime, Beijing, China), proteins in each cell lysate were quantified using a Pierce^TM^ BCA Protein Assay Kit (Thermo Fisher Scientific, Waltham, MA, USA), mixed with 2X Laemmli SDS-PAGE sample buffer then separated using 12% SDS-PAGE and transferred onto a PVDF membrane (Millipore, Billerica, MA, USA). After blocking with PBS containing 5% skim milk, the membrane was probed with mouse anti-pCD163 monoclonal antibody (AbD Serotec, Oxford, England) or 6D10. Specific binding of antibodies to their targets was detected by horseradish peroxidase (HRP)-conjugated goat anti-mouse IgG (Jackson ImmunoResearch, West Grove, PA, USA) and revealed using ECL chemiluminescence substrates (Bio-Rad, Hercules, CA, USA). The chemiluminescence signal was digitally recorded using a ChemiDoc^TM^ MP Imaging System (Bio-Rad). The membrane was also probed with mouse anti-GAPDH monoclonal antibody (Sigma-Aldrich, St. Louis, MO, USA) for protein loading normalization.

### Immunofluorescence (IFA)

MH-S^CD163^ and RAW264.7^CD163^ cells were seeded onto coverslips and separately infected with various PRRSV strains. Next, cells were fixed with 4% paraformaldehyde and permeabilized with PBS containing 0.5% Triton X-100 (Sigma-Aldrich). After blocking with PBS containing 1% BSA, coverslips were probed with mouse anti-porcine CD163 monoclonal antibody (AbD Serotec) or 6D10. Specific antibody binding was detected using Alexa Fluor^TM^ 488-conjugated goat anti-mouse IgG (Thermo Fisher Scientific) and coverslips were mounted onto slides using Prolong Gold antifade reagent containing 4’6-diamidino-2-phenylindole (DAPI) (Thermo Fisher Scientific) for visualization using a Leica AF6000 fluorescence microscope (Leica, Germany).

### RNA isolation and quantitative real-time PCR (qPCR)

Total RNA was isolated from MH-S^CD163^ or RAW264.7^CD163^ cell lines or PAMs infected with PRRSV at the indicated time points using RNAiso Plus (TaKaRa, Dalian, China) and reverse transcribed using the PrimeScript® RT reagent Kit (TaKaRa) according to the manufacturer’s instructions. The qPCR was performed in duplicates using the StepOne Plus® Real-Time PCR System (Applied Biosystems, Foster City, CA, USA) using FastStart Universal SYBR Green Master (Roche). The equal expression of GAPDH from these cells was confirmed and used to normalize the total amount of input RNA. The related expression levels of indicated genes were quantified by the 2^-∆∆CT^ method as previous described [40]. The qPCR primers and their corresponding sequences are listed in Table 1 and their efficiency was validated.

### Statistical analysis

Statistical significance was assessed using paired two-tailed Student’s *t* test with PRISM software (Version 6; GraphPad software). A *P*-value of less than 0.05 was considered to be statistically significant.

## Results

### Generation and characterization of MH-S^CD163^ and RAW264.7^CD163^ cell lines

**Fig. 1:**
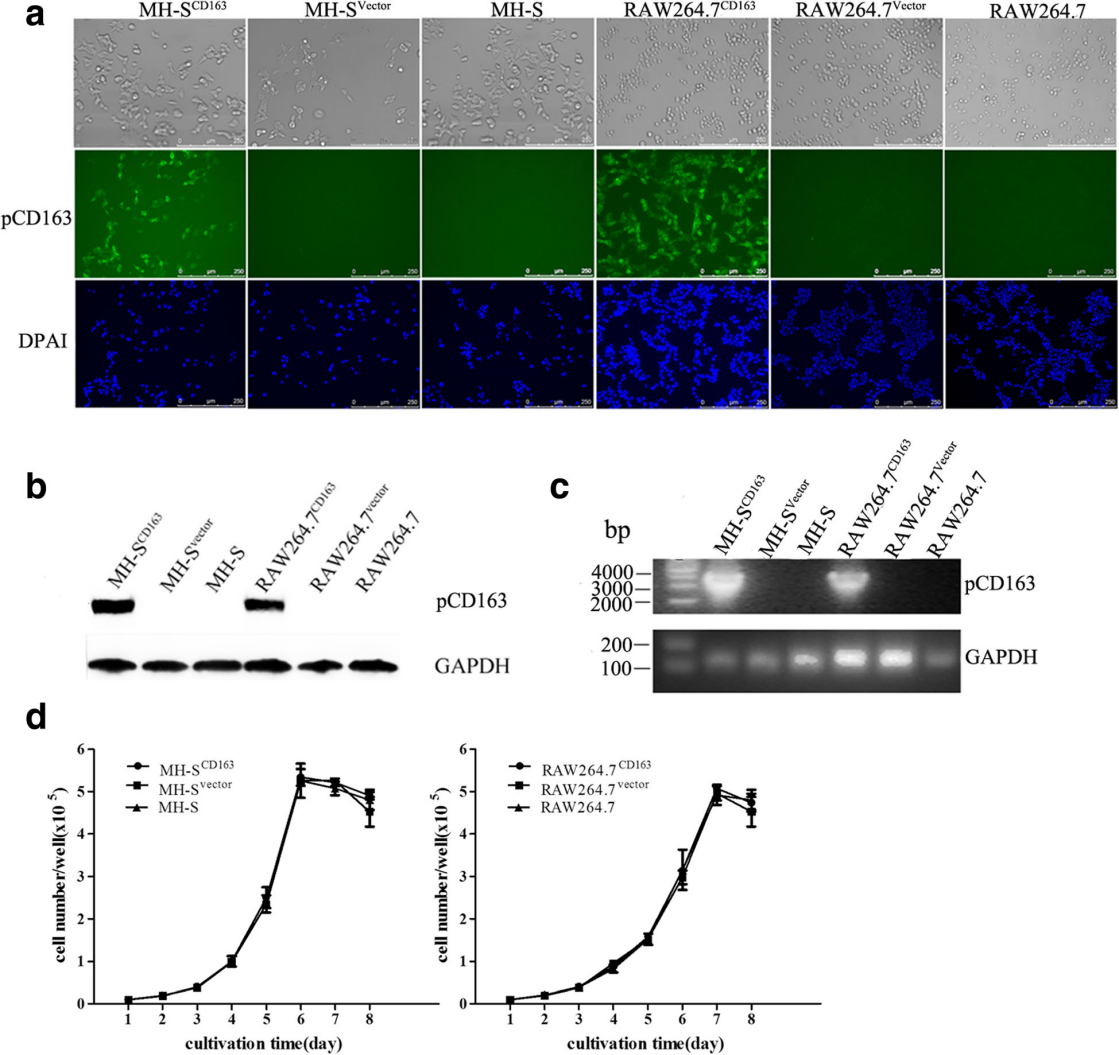
Generation of MH-S^CD163^ and RAW264.7^CD163^ cell lines. MH-S and RAW264.7 cells were transduced with the indicated lentiviral constructs and puromycin-resistance cells were selected and subcloned. Each cell line was stained with anti-pCD163 mAb to measure pCD163 expression by immunofluorescence assay (**a**) and western blot (**b**). **c** Total RNA of each cell line was isolated and reverse transcribed to amplify the full-length gene coding for pCD163. GAPDH transcripts were amplified to normalize the total amount of input RNA. **d** The growth curves of cells transduced with the indicated lentivirus are shown. Cells for each clone were seeded at a concentration of 1×10^4^ cells/well and split daily for eight consecutive days and half of the cells were counted to determine cell number. The average cell count for each clone at each time point was plotted against time. Values indicate the mean ± SD from three replicates

MH-S^CD163^ and RAW264.7^CD163^ cell lines were generated after transduction of recombinant lentivirus encoding pCD163 followed by puromycin selection. In both cell lines, the exclusive expression of pCD163 was demonstrated by IFA and western blot; negative expression was observed in controls including parental cells and cells transduced with empty lentiviral vector (Fig. 1a and b). Expression of pCD163 was also supported by the presence of the full-length gene encoding pCD163, as determined by RT-PCR analysis (Fig. 1c).

To evaluate the effect of pCD163 expression on the growth characteristics of MH-S^CD163^ and RAW264.7^CD163^ cell lines, growth curves of both cell lines expressing pCD163 were grown in parallel with the parental cells and cells transduced with empty vector. Cells were initially seeded at a density of 1×10^4^ cells/well and every 24 h cultured cell numbers were counted. As shown in Fig. 1d, no differences in cell numbers were observed among pCD163-expressing cells, corresponding parental cells and empty vector-transduced cells.

### MH-S^CD163^ and RAW264.7^CD163^ cells are susceptible to PRRSV infection

**Fig. 2:**
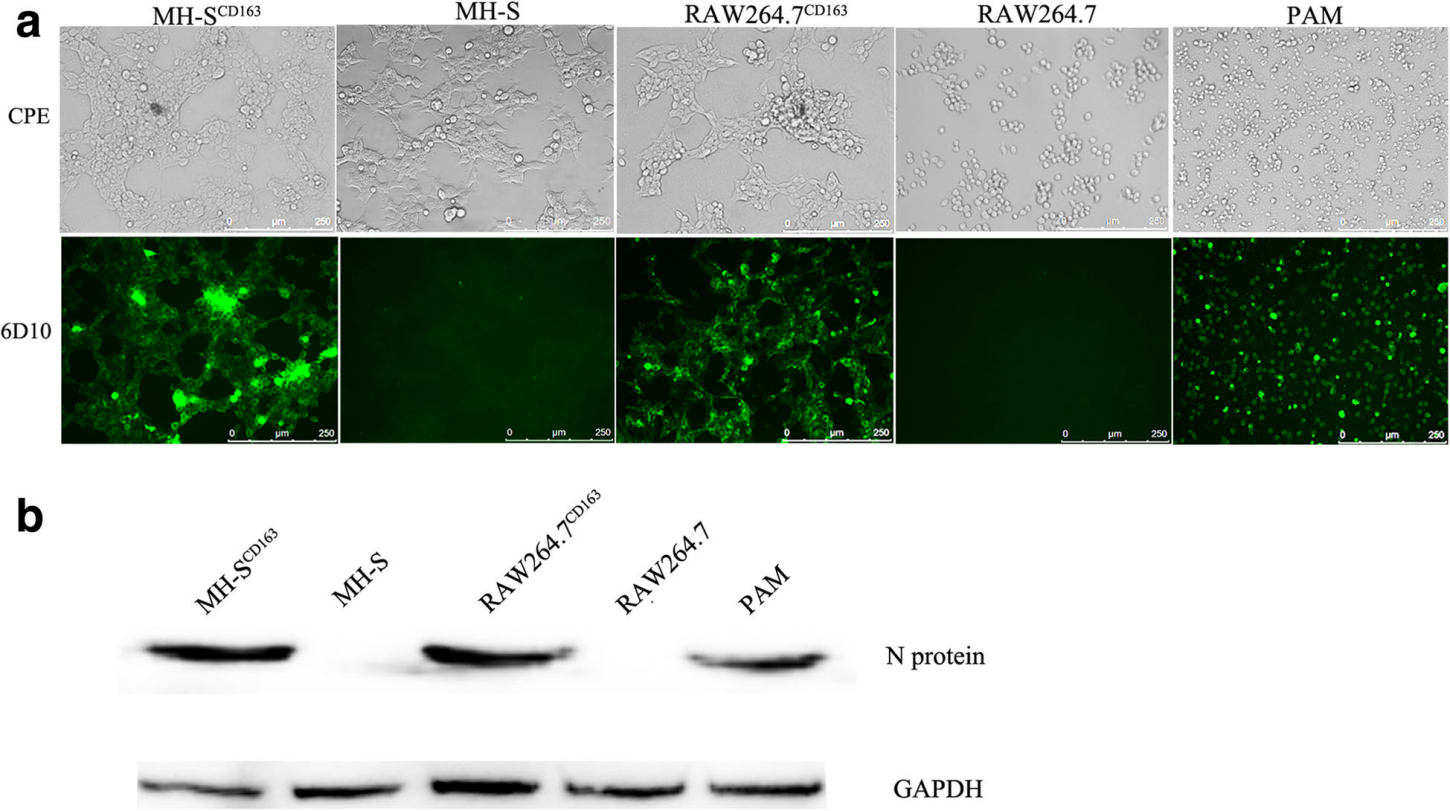
PRRSV infections in MH-S^CD163^ and RAW264.7^CD163^ cell lines. The MH-S and RAW264.7 cell lines and PAMs were inoculated with JXA1 at 1 MOI. **a** CPEs were visualized at 24 hpi using an inverted microscope (200×). Meanwhile, cells were fixed and permeabilized to measure virus infection using immunofluorescence staining of virus using anti-PRRSV N protein-specific mAb (6D10). Images are representative one of three independent experiments. **b** Cell infection was detected using anti-PRRSV N protein-specific mAb labeling of western blot using GAPDH as the control

MH-S^CD163^, RAW264.7^CD163^, parental cells and PAMs were inoculated with the PRRSV JXA1 strain at 1 MOI and PRRSV infection of these cells was determined. As shown in Fig. 2a, a typical cytopathic effect (CPE) was observed in PRRSV-infected MH-S^CD163^ and RAW264.7^CD163^ cells. Moreover, in contrast to undetectable N protein levels in either MH-S or RAW264.7 cells after incubation with PRRSV for 24 h, MH-S^CD163^ and RAW264.7^CD163^ cells and PAMs each demonstrated N protein detection by IFA (Fig. 2a) and western blot (Fig. 2b).

### MH-S^CD163^ and RAW264.7^CD163^ cells support replication of various genotype 2 PRRSV isolates

**Fig. 3:**
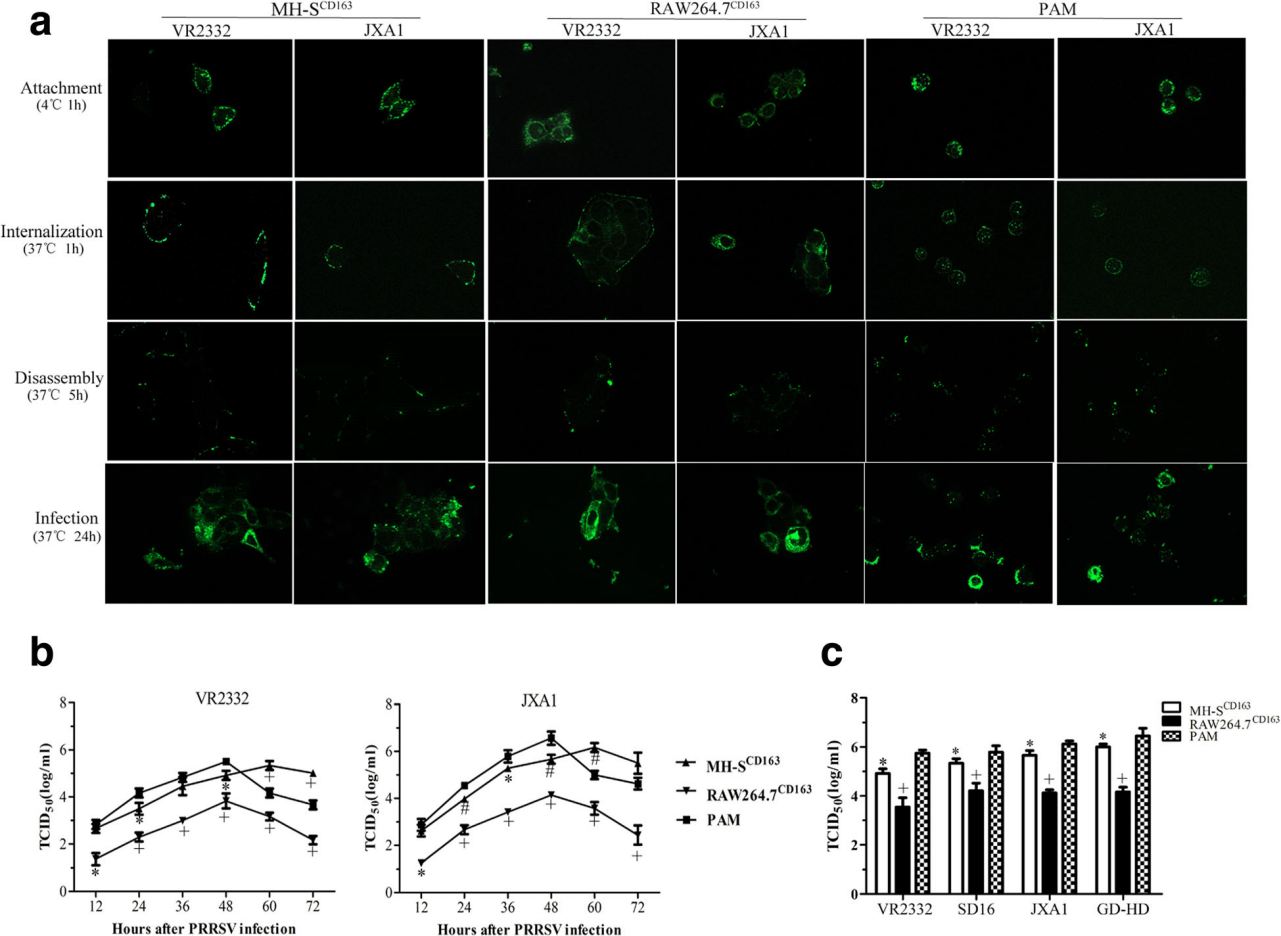
Susceptibility of MH-S^CD163^ and RAW264.7^CD163^ cell lines to various genotype 2 PRRSV isolates. **a** MH-S^CD163^ and RAW264.7^CD163^ cell lines and PAMs were inoculated with JXA1 and VR-2332 at 10 MOI. Various sequential stages of the viral replication cycle were measured by immunofluorescence staining of virus using anti-PRRSV N protein-specific mAb. Images are representative of three independent experiments. **b** PRRSV replication in MH-S^CD163^ and RAW264.7^CD163^ cells. The MH-S^CD163^ cells (triangle), RAW264.7^CD163^ cells (inverted triangle **)** and PAMs (square) were inoculated with JXA1 and VR-2332 at 0.1 MOI. The lysate of each infected cell line at the indicated time points was collected and titrated on MARC-145. Values represent the mean ± SD from three independent experiments. *, *P* < 0.05; ^#^, *P* < 0.01; ^+^, *P* < 0.001. **c** The MH-S^CD163^ and RAW264.7^CD163^ cells, parental cell lines and PAMs were inoculated with various PRRSV isolates (VR-2332, SD16, JXA1 and GD-HD) at 0.1 MOI. The lysate of each cell line infected with each indicated PRRSV isolate was collected at 48 hpi and titrated on MARC-145 cells. Values indicate the mean ± SD from three independent experiments. *, *P* < 0.05; ^#^, *P* < 0.01; ^+^, *P* < 0.001

We next analyzed the PRRSV replication cycle in MH-S^CD163^ and RAW264.7^CD163^ cells. Both cell lines were inoculated with either JXA1 or VR-2332 at a MOI of 10 and PRRSV-infected PAMs served as the positive control. The PRRSV-N protein was visualized using anti-N protein mAb to monitor different stages of the PRRSV replication cycle. As shown in Fig. 3a, PRRSV attachment, internalization, disassembly and infection stages were all observed in the MH-S^CD163^, RAW264.7^CD163^ cells and PAMs. The attachment of virus particles to cell surface was observed first after incubation of cells with virus at 4°C. Next, virus particles were internalized into cells when the incubation temperature was shifted to 37°C. As virus particles were disassembled within cells, PRRSV-specific proteins were not clearly detected in infected MH-S^CD163^ and RAW264.7^CD163^ cells. As assembly of PRRSV virions was eventually completed, PRRSV-N-positive cell staining was again observed in MH-S^CD163^ and RAW264.7^CD163^ cells at 24 hpi (Fig. 3a).

To further evaluate the replication efficiency of PRRSV in MH-S^CD163^ and RAW264.7^CD163^ cell lines relative to PAMs, the cell lines were separately infected with PRRSV strains VR-2332 and JXA1 at 0.1 MOI and the virus yields at indicated time points were measured by calculating TCID_50_. As shown in Fig. 3b, no significant difference was observed for either VR-2332 or JXA1 viral titers in MH-S^CD163^ cell lines at 12 hpi; however, from 24 hpi to 48 hpi significantly lower VR-2332 or JXA1 viral titers were observed in MH-S^CD163^ cells than in PAMs (*p* < 0.05), with the exception of VR-2332 at 36 hpi. Titers observed in the RAW264.7^CD163^ cell line were lowest (*p* < 0.001), while titers at 60 hpi and 72 hpi were significantly higher in MH-S^CD163^ cells than in PAMs (*p* < 0.01), with the exception of JXA1 at 72 hpi. Furthermore, a 12-h lag time to attain peak viral titers was observed for MH-S^CD163^ cells compared with the time of peak viral titer for PAMs; however, no significant difference in peak titers was observed between MH-S^CD163^ cells at 60 hpi and PAMs at 48 hpi.

We further evaluated the replication efficiency of various genotype 2 PRRSV isolates in MH-S^CD163^ and RAW264.7^CD163^ cells. In agreement with the results in Fig. 3b, the viral titers of all genotype 2 PRRSV isolates (VR-2332, SD16, JXA1 and GD-HD) in MH-S^CD163^ cells were significantly lower than that in PAMs at 48 hpi (*p* < 0.05), while viral titers in RAW264.7^CD163^ were the lowest (*p* < 0.001) (Fig. 3c). Collectively, PRRSV replication efficiency in MH-S^CD163^ cells was more efficient than in RAW264.7^CD163^ cells and was more similar to that observed in PAMs.

### Cytokine expression patterns in MH-S^CD163^ and RAW264.7^CD163^ cell lines after PRRSV infection

**Fig. 4:**
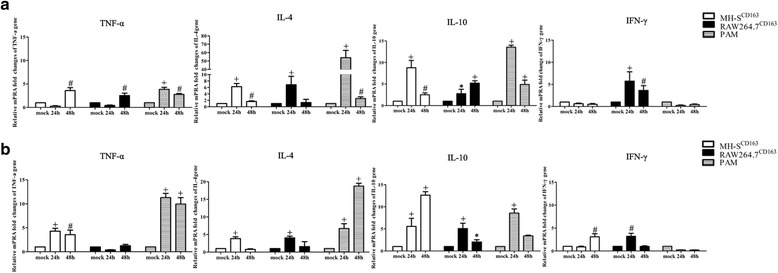
Cytokine expressions in MH-S^CD163^ and RAW264.7^CD163^ cell lines. Abundance of cytokine mRNAs in MH-S^CD163^, RAW264.7^CD163^ cells or PAMs inoculated with JXA1 (**a**) and VR-2332 (**b**) was determined by qPCR. Values were normalized to corresponding uninfected MH-S^CD163^, RAW264.7^CD163^ cells or PAMs at 24 hpi, respectively, and indicate the mean ± SD from three replicates. *, *P* < 0.05; ^#^, *P* < 0.01; ^+^, *P* < 0.001

The expression levels of tumor necrosis factor-alpha (TNF-α), IL-4, IL-10 and interferon-γ (IFN-γ) in MH-S^CD163^ and RAW264.7^CD163^ cells after PRRSV infection at 24 hpi and 48 hpi were measured and the results are shown in Fig. 4a (JXA1 infection) and Fig. 4b (VR-2332 infection). In JXA1-infected MH-S^CD163^ cells, a similar pattern of a significant increase of IL-4 and IL-10 mRNA expression at 24 hpi followed by a decrease at 48 hpi was observed and mirrored the expression pattern in PAMs (*p* < 0.01). Meanwhile, TNF-α mRNA level decreased at 24 hpi and increased at 48 hpi in JXA1-infected MH-S^CD163^ cells. IFN-γ mRNA in both JXA1-infected MH-S^CD163^ cells and PAMs exhibited for a low basal level of expression. In JXA1-infected RAW264.7^CD163^ cells, the highest mRNA expression of IL-4 and IFN-γ at 24 hpi and TNF-α and IL-10 at 48 hpi were observed (Fig. 4a). In Fig. 4b, the mRNA expression level of TNF-α in MH-S^CD163^ cells was similar to that for PAMs after VR-2332 infection. A significant up-regulation of mRNA expression levels of IL-4 and IL-10 was observed in MH-S^CD163^ and RAW264.7^CD163^ cells at 24 hpi (*p* < 0.001) relative to mock infected controls, which mirrored the pattern observed in PAMs. Moreover, a significant increase of IFN-γ mRNA was detected in MH-S^CD163^ at 48 hpi and RAW264.7^CD163^ cells at 24 hpi (*p* < 0.01). Taken together, the characteristics of cytokine expression in MH-S^CD163^cells were more similar to expression patterns in PAMs as compared to patterns observed in RAW264.7^CD163^ cells.

## Discussion

*In vitro*, efficient PRRSV replication can only be observed in porcine-derived primary cells (e.g., PAMs) and MARC-145 cells. Moreover, PK-15, BHK-21 and CRL-2843 cell lines expressing pCD163 were also used in some cases for PRRSV infection. However, due to the lack of designation of novel porcine origin immunologic molecules as human or mouse counterpart and corresponding antibodies, studies on PRRSV pathogenesis and immune responses in swine-derived primary cells or cells lines have been significantly hampered. Considering that these two murine cell lines used in this study were derived from murine macrophage lineage which classically designated to be involved in innate and/or adaptive immune responses, it would be critical to investigate whether they may further mimic the natural host PAMs for PRRSV infection *in vitro*. Therefore, we developed MH-S^CD163^ and RAW264.7^CD163^ cell lines stably expressing pCD163 that support PRRSV infection with similar features as that observed in PAMs.

CD163 is a group B cysteine–rich scavenger receptor expressed exclusively in cells of the monocyte-macrophage lineage [41]. As a type I membrane protein, the extracellular region of CD163 contains nine scavenger receptor cysteine-rich (SRCR) domains (SRCR1-9) and is anchored by a single transmembrane portion and a short cytoplasmic domain [42, 43]. Biologically, membrane-associated CD163 is responsible for mediating endocytosis of hemoglobin-haptoglobin complexes to prevent tissue damage caused by free hemoglobin-catalyzed production of iron-derived hydroxyl radicals [44–46] and modulating systemic immune homeostasis, particularly with respect to anti-inflammation [47]. In *in vivo* study, alveolar macrophages from asthmatic patients with reduced cell-surface expression of CD163 associated with inflammatory effects, which was similar as that observed in CD163 knock-out mice [47]. CD163 also serves as a receptor for erythroblasts, bacteria and viruses [48–50]. Increasing evidence has demonstrated that pCD163 is identified as a fusion receptor for PRRSV[25, 43], a further study on generation of genome edited pigs with the deletion of SRCR5 confirmed SRCR5 of pCD163 was essential for successful infection with PRRSV, more importantly, there was no other biological function of pCD163 affected[51]. Pro-inflammatory and anti-inflammatory effects caused by virus infection were demonstrated to associate with CD163 expressed on immune cells [52]. However, regarding pCD163, whether or not it plays the same role as its human counterpart remains to be elucidated; as yet we may not rule out the possibility of other SRCR domains involved in its other biological functions.

From the study of van Breedam’s group, non-permissive BHK21 cells were found to be rendered susceptible to PRRSV infection with transfection of CD163 cDNAs from various species (e.g., human-, monkey-, murine- and porcine-origin), [53]. While these observations partially explain the susceptibility of CD163-transfected Marc145 cells to PRRSV *in vitro*, they do not explain why swine and related species of wild boar are the only known *in vivo* hosts of PRRSV. Moreover, although truncation assays have demonstrated that SRCR5 of pCD163 mediates PRRSV infection [54], replacement of the SRCR5 domain of pCD163 with the SRCR5 domain from the human CD163-like homolog (CD163Li) only conferred resistance to genotype 1 PRRSV, not genotype 2 virus [55]. Therefore, mechanisms of CD163 involvement in PRRSV infection are still not fully understood and the roles played by CD163 from various species in PRRSV infection remain to be elucidated. Since knockout of pCD163 confers complete resistance to PRRSV in swine, it would be interesting to know whether replacement of murine CD163 with porcine CD163 could render mice susceptible to PRRSV.

Based on our data, expression of endogenous murine CD163 in both parental cell lines was undetectable at the protein level (Additional file 1: Figure S1), which is consistent with findings of a previous study showing expression of murine CD163 at a low basal level in RAW264.7 cells using qPCR [56]. Moreover, analyses of PRRSV replication and PRRSV-N protein expression have indicated that parental MH-S and RAW264.7 cells are not susceptible to PRRSV infection.

According to our results, MH-S^CD163^ cells may be more susceptible to PRRSV infection due to significantly higher virus titers in MH-S^CD163^ cells compared with that observed in RAW264.7^CD163^ cells. Furthermore, we found significantly lower viral titers of all genotype 2 PRRSV isolates were in MH-S^CD163^ cells at 48 hpi compared with titers in PAMs (Fig. 3c), probably due to a 12-h lag time to reach peak viral titers in MH-S^CD163^ cells in comparison to PAMs (Fig. 3b).

The poor adaptive immune response to PRRSV in piglets has been partially ascribed to abnormal up-regulation of IL-10 [57]. Moreover, PRRSV-induced IL-10 production has been reported to be associated with low levels of IFN-γ production in infected cells [58, 59]. In our study, up-regulation of IL-10 and down-regulation of IFN-γ were observed in PRRSV-infected MH-S^CD163^ cells, which is consistent with data regarding cytokine production by PAMs.

## Conclusion

Two PRRSV-susceptible murine macrophage-derived cell lines were established by introducing pCD163 in MH-S and RAW264.7 cells. Consequently, the MH-S^CD163^ cell line was shown to have greater investigational value for further study of immune responses after PRRSV infection, since its PRRSV susceptibility and mRNA cytokine expression levels were relatively similar to those observed for primary PAMs. Our research thus provides mouse macrophage cell models which may mimic natural host cells *in vitro* to aid the study of PRRSV pathogenesis and immune response mechanisms to viral infection.

## Additional file

Additional file 1: Figure S1. Endogenous mCD163 expression in MH-S and RAW264.7 cell lines. MH-S and RAW264.7 cell lysates were separated by SDS-PAGE and proteins were transferred to PVDV membrane and probed with anti-pig CD163 SRCR1-4 polyclonal antisssbody that cross reacts with mCD163. Mouse liver tissue lysate served as the positive control by western blot using GAPDH as the internal protein control. (JPEG 8 kb)

## Abbreviations

BHK-21: baby hamster kidney cells
CD: cluster of differentiation
CPE: cytopathic effect
DAPI: 4’6-diamidino-2-phenylindole
DMEM: Dulbecco’s Modified Eagle Medium
FBS: fetal bovine serum
HRP: horseradish peroxidase
HS: heparin sulfate
IFA: Immunofluorescence assay
IFN-γ: interferon-γ
MOI: multiplicity of infection
MuLV: murine leukemia virus
ORFs: open reading frames
PAMs: porcine alveolar macrophages
pCD163: porcine CD163
pi: post-infection
PK-15: porcine kidney cell line
PRRSV: porcine reproductive and respiratory syndrome virus
qPCR: quantitative real-time PCR
SDS-PAGE: Sodium dodecyl sulfate-polyacrylamide gel electrophoresis
SRCR: scavenger receptor cysteine-rich
TCID_50_: the median tissue culture infective dose
TNF-α: tumor necrosis factor-alpha
