# Supplementary figures and images for: Generation of murine macrophage-derived cell lines expressing porcine CD163 that support porcine reproductive and respiratory syndrome virus infection
Source: BMC Biotechnol. 2017 Nov 9;17:77. doi: 10.1186/s12896-017-0399-5 (PMC5680797; doi:10.1186/s12896-017-0399-5)

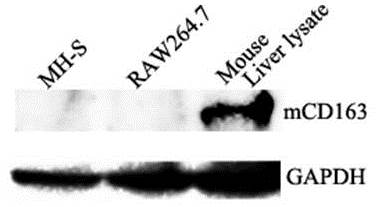

Supplement: Additional file 1: Figure S1. — Endogenous mCD163 expression in MH-S and RAW264.7 cell lines. MH-S and RAW264.7 cell lysates were separated by SDS-PAGE and proteins were transferred to PVDV membrane and probed with anti-pig CD163 SRCR1-4 polyclonal antisssbody that cross reacts with mCD163. Mouse liver tissue lysate served as the positive control by western blot using GAPDH as the internal protein control. (JPEG 8 kb) [file 12896_2017_399_MOESM1_ESM.jpg]
